# Health-related quality of life of children and adolescents with type 1 diabetes during the COVID-19 pandemic in Kuwait

**DOI:** 10.3389/fpubh.2022.1056967

**Published:** 2022-12-23

**Authors:** Dalia Al-Abdulrazzaq, Doaa Khalifa, Taiba Alqaisi, Fatima Al-Juailla, Fouzeyah Othman, Sarah Qabazard, Hessa Al-Kandari

**Affiliations:** ^1^Department of Pediatrics, Faculty of Medicine, Kuwait University, Kuwait City, Kuwait; ^2^Department of Population Health, Dasman Diabetes Institute, Kuwait City, Kuwait; ^3^Department of Pediatrics, Al-Farwaniya Hospital, Kuwait City, Kuwait

**Keywords:** children, type 1 diabetes, health-related quality of life, PedsQL^TM^ Diabetes Module, COVID-19

## Abstract

**Background:**

With the rapid transmission of COVID-19 globally, countries have implemented strict governmental measures and social distancing rules that aimed to minimize the spread of the virus. School closures, quarantine orders, and social isolation, coupled with a surge in family stress and lack of peer interactions, are probable causes of mental health complications and psychological symptoms faced by children. This study aims at comparing the HRQoL of children and adolescents with type 1 diabetes (T1D) and affected by COVID-19 infection (personal or familial) and those who were not affected by COVID-19.

**Materials and methods:**

A random sample was selected from children and adolescents diagnosed with T1D at the six major governmental diabetes centers in Kuwait. To measure HRQoL, parent-proxy and self-reports from the Pediatric Quality of Life Inventory (PedsQL^TM^) 3.0 Diabetes Module were used.

**Results:**

A sample of 455 children and adolescents with T1D diabetes (44.6% male participants and 41.98% affected by COVID-19 infection) was studied. The total score of the HRQoL self-reports was significantly higher compared with parent-proxy reports (79.06 ± 15.19 vs. 73.79 ± 15.17, *p* < 0.01). Children reported significantly higher HRQoL scores in the “treatment I” domain and “worry” domain and lower scores in the “diabetes” symptoms domain, compared with their parents' reports (*p* < 0.01). In the COVID-19-affected group, a major difference was noticed between the total scores of children and parent-proxy reports (77.04 ± 15.81 vs. 72.80±14.90, *p* = 0.047). The affected children reported significantly lower scores in “diabetes” symptoms (59.50) (*p* < 0.01) and higher scores in the “treatment I” domain (81.43) than their parent-proxy reports (72.05) (*p* < 0.01).

**Conclusion:**

This is the first report on the health-related quality of life of children with T1D in Kuwait during the COVID-19 era. Parents' or caregivers' experience of caring for their children was negative, as they worried, and reported poorer HRQoL compared with their children's perceptions. There is a need to empower healthcare professionals to support parents and caregivers of children with chronic diseases such as T1D in promoting self-management, enhancing physical and psychological wellbeing, treatment adherence, and continuous health education during pandemics of any kind.

## Introduction

The COVID-19 pandemic has undoubtedly impacted all areas of the global population's daily lives, both physically and mentally, making it a worldwide healthcare disaster. With the rapid transmission of COVID-19 globally, countries have implemented strict governmental measures and social distancing rules that aimed to minimize the spread of the virus. School closures, quarantine orders, and social isolation, coupled with a surge in family stress and lack of peer interactions, are probable causes of mental health complications and psychological symptoms faced by children (1, 2). A recent meta-analysis using data from 80,879 youths globally, to investigate the pandemic's effect on mental health, reported a pooled prevalence estimate of clinically elevated depression and anxiety: 25.2% for children and 20.5% for adolescents. Compared with pre-pandemic estimates, the prevalence of depression and anxiety symptoms during COVID-19 has doubled (1). Mental health problems, particularly hyperactivity and peer problems, as well as psychosomatic complaints, such as sleeping problems and headaches, were also experienced by children during the pandemic (3).

Children with chronic illnesses faced additional challenges during the COVID-19 pandemic, including hindrance to accessing inpatient and outpatient clinical care. These limitations in care may have negatively impacted children with type 1 diabetes (T1D) since diabetes pediatric centers shifted to telemedicine (4). Families with children and adolescents with T1D were forced to change their approach to disease management as health services and delivery became significantly disrupted (4, 5). This may have resulted in delayed diabetes diagnosis, delayed insulin delivery, more episodes of diabetic ketoacidosis (DKA) and hyperglycemia, and admission to the pediatric intensive care unit (PICU); families also experienced fear of contracting COVID-19 infection while obtaining care (6). Furthermore, COVID-19 infection itself in individuals with preexisting diabetes has been associated with higher rates of hospitalization and admission to the intensive care unit (7, 8). These might indeed have a negative impact on an infected child's mental health and wellbeing. It might be also safe to assume that COVID-19 infection in a close family member might have a negative impact on the child, as the role of family dynamics and functioning influence diabetes management behavior and metabolic control (9).

Within and beyond the context of the pandemic, children with T1D face daily challenges, such as intensive therapeutic insulin regimes, dietary restrictions, and necessary physical activity (10). These children are also prone to the development of profound feelings of uncertainty, fear, or irritability that are a result of home confinement and disruptions in normal routine, which may ultimately influence their diabetes management (4, 11). These factors in combination make it imperative to address the emotional state of this population and assess their health-related quality of life (HRQoL) during the pandemic.

The assessment of HRQoL is crucial in clinical trials and healthcare as it measures the impact of diseases or disabilities on the mental, physical, and social dimensions of a patient's health (12, 13). Diabetes-specific HRQoL measurement instruments are critical for the assessment of symptoms and problems relevant to people living with diabetes, as well as the person's adherence to diabetes self-management (14, 15). Considering the obstacles of accessing healthcare and diabetes management as well as the emotional consequences resulting from the COVID-19 pandemic, it may be expected that pediatric patients with T1D have impaired HRQoL.

One objective of this study was to compare the HRQoL of children and adolescents (aged 2–18 years) with T1D and affected by COVID-19 infection (personal or familial) and those who were not affected by COVID-19. An additional objective is to measure the similarities and differences between the children's and parents' HRQoL reports during the COVID-19 pandemic.

## 2. Methods

### 2.1. Study design and subjects

Starting in January 2021, a random sample was selected from children and adolescents diagnosed with T1D between January 2011 and January 2020 at the six major governmental diabetes centers in Kuwait. Children aged 2–18 years who met the inclusion criteria: diabetes duration for at least 1 year, resident of Kuwait during the pandemic for at least 6 months, and Arabic-speaking parents and children, were included. Participants with other comorbidities (such as developmental delay and chronic renal disease) apart from T1D and its complications, and parents who refused to participate were excluded.

A total of 656 families were approached; of these, 586 (89.3%) were eligible while 70 (10.6%) did not meet the inclusion criteria; among those eligible, 455 (77.6%) agreed to participate. Of the 455 families, 116 fathers, 334 mothers, and 3 legal guardians were interviewed. A total of 203 boys and 252 girls were enrolled, including 49 children aged 2–4 years, 74 children aged 5–7 years, 204 children aged 8–12 years, and 128 children aged 13–18 years at the time of interview; these age groups correspond with the HRQoL instrument's age categories.

### 2.2. Data collection

Interviews were performed from January to October 2021, after parental consent and child assent were obtained. Most families participated over the phone or *via* a web-based platform (*n* = 433), but 22 families were directly interviewed during clinical visits. The PedsQL^TM^ 3.0 Diabetes Module Arabic version, a validated questionnaire for children, was used to assess diabetes-specific HRQoL (16, 17). Before completion of the PedsQL^TM^ 3.0 forms, baseline data were retrieved from parents or caregivers regarding demographic characteristics, diabetes-related history, and COVID-19 infection history. Glycemic status was obtained from medical records in terms of glycated hemoglobin (HbA1c) within 3 months of the recruitment. Optimal control was defined as HbA1c of < 7% as per the 2018 International Society of Pediatric and Adolescent Diabetes (ISPAD) guidelines, and HbA1C of above 9.0% was considered high risk (18). The study protocol was approved by the Standing Committee for Coordination of Health and Medical Research (Ethics Review Committee) at the Ministry of Health (MoH) of Kuwait (Reference No. 1588/2020).

### 2.3. PedsQL^TM^ 3.0 Diabetes module

The 28-item module is composed of five scales: (1) diabetes symptoms (11 items), (2) treatment barriers I (4 items), (3) treatment adherence II (7 items), (4) worry (3 items), and (5) communication (3 items). The module is designed for four age groups: parent-proxy reports for toddlers (2–4 years), parent-proxy and child self-reports for young children (5–7 years), school-age children (8–12 years), and teenagers (13–18 years). The PedsQL^TM^ items follow the five-point Likert scale from 0 (never) to 4 (almost always) for all age groups, except for young children (aged 5–7 years), which follows a three-point scale: 0 (not at all), 2 (sometimes), and 4 (a lot). To calculate the health-related quality of life (HRQoL), items were reverse-scored and linearly transformed on a scale from 0 to 100 (0 = 100, 1 = 75, 2 = 50, 3 = 25, and 4 = 0), and the sum of all items was divided over the number of items answered to calculate the total score. Higher scores indicate better HRQoL (19, 20).

The original PedsQL^TM^ 3.0 Diabetes module was translated into Arabic and validated on children with T1D and their parents/caregivers at Kuwait University, Kuwait, based on the linguistic validation guidelines of the PedsQL^TM^ Quality of Life Questionnaire and approved by MAPI Research Trust, on behalf of Dr. James W. Varni, the copyright owner of PedsQL^TM^ (17, 21).

### 2.4. Procedure

In preparation for the study, a comprehensive well-established data collection sheet was developed. Research assistants were trained as interviewers. Following patient identification, a pilot phase was implemented over a 1-month period to test the procedures with a convenient sample of 50 families. The interview process and data collection forms were adjusted after the pilot phase.

The interviews took 20–25 min. The survey consisted of five sections: personal information, diabetes history, COVID-19-related history, COVID-19 family history, and the PedsQL 3.0 questionnaires. The answers were documented by the interviewers based on parents'/children's responses.

To ensure proper enrollment, COVID-19 status in children was confirmed through the Pediatric COVID-19 Registry (PCR-Q8) in Kuwait (MoH Reference No. 2020/1233), a national registry that records all cases of severe acute respiratory syndrome coronavirus 2 (SARS-CoV-2) in children in the country since the start of the pandemic in February 2020.

### 2.5. Statistical analysis

Statistical analysis was conducted using STATA software 13.1 (STATA Corp, College Station, TX). Continuous variables were expressed as median and interquartile ranges (IQR), as they were non-normally distributed. The Kruskal–Wallis test was used to test for differences in the continuous variables. The HRQoL scores were expressed as mean and standard deviation (SD). Unpaired *t*-tests were used to compare total and dimension-specific HRQoL scores between groups (e.g., no COVID-19 infection vs. COVID-19 infection).

## 3. Results

A total of 455 children and adolescents [203 (44.6%) male participants and 252 (55.4%) female participants] with T1D were included. The baseline characteristics of study participants and COVID-19 infection status are shown in Table 1. Among the participants, 191 (41.98%) children were affected by COVID-19. Children who had had COVID-19 were significantly older at the time of the study than those who had not (11.5 vs. 10.7 years, *p* = 0.035). The majority (31.8%) of the COVID-19-affected children were from families with incomes of 2,000 KD (equivalent to approximately €6,000) and above (*p* = 0.031). The majority (76.9%) of the children affected by COVID-19 had glycemic control in the high-risk category (*p* = 0.018), with a significantly higher HbA1c than those who had not been infected with COVID-19 [10.1 (9.1;11.6) vs. 9.4 (8.2;10.8), *p* < 0.01]. Children who had and had not been affected by COVID-19 were not different in terms of gender, parents' education, household dynamics, diabetes duration, insulin regimen, or family history of T1D. HRQoL was collected for 620 participants based on 165 child self-reports and 455 parent-proxy reports from the PedsQL™ 3.0 Diabetes module. A total of 260 participants were affected by COVID-19, of which 69 were children and 191 were parents. A total of 360 participants not affected by COVID-19 responded to the HRQoL questionnaire, of which 96 were children and 264 were parents.

**Table 1:** Baseline characteristics of children with T1D at HRQoL assessment.

	**Total**	**Affected by COVID-19**	**Unaffected by COVID-19**	***p***
	**(*****n*** = **455)**	**(*****n*** = **191, 41.98%)**	**(*****n*** = **264, 58.02%)**	
	***n (%)***	***n (%)***	***n (%)***	
**Age at interview**				
2–4	49 (10.8)	15 (7.9)	34 (12.9)	0.359
5–7	74 (16.3)	31 (16.2)	43 (16.3)	
8–12	204 (44.8)	87 (45.5)	117 (44.3)	
13–18	128 (28.1)	58 (30.4)	70 (26.5)	
*Median (IQR)*	11.0 (7–13)	11.5 (8.3–13.5)	10.7 (7.2–13.1)	0.035^*^
**Gender**				
Male	203 (44.6)	75 (39.3)	128 (48.5)	0.051
Female	252 (55.4)	116 (60.7)	136 (51.5)	
**Father education level**				
None	0 (0.0)	0 (0.0)	0 (0.0)	0.461
Primary	5 (1.3)	2 (1.2)	3 (1.3)	
Intermediate	41 (10.5)	19 (11.7)	22 (9.6)	
Secondary	92 (23.5)	44 (27.2)	48 (20.7)	
Diploma	70 (17.9)	24 (14.8)	46 (20.1)	
University degree or above	183 (46.8)	73 (45.1)	110 (48.0)	
**Mother education level**				
None	3 (0.8)	2 (1.2)	1 (0.4)	0.932
Primary	2 (0.5)	1 (0.6)	1 (0.4)	
Intermediate	38 (9.6)	16 (9.6)	22 (9.6)	
Secondary	64 (16.2)	29 (17.5)	35 (15.3)	
Diploma	92 (23.3)	39 (23.5)	53 (23.1)	
University degree or above	196 (49.6)	79 (47.6)	117 (51.1)	
**Total family income (KD)**				
< 1,000	104 (24.7)	35 (19.9)	69 (28.2)	0.031^*^
1,000–1,499	89 (21.1)	31 (17.6)	58 (23.7)	
1,500–1,999	54 (12.8)	23 (13.1)	31 (12.6)	
2,000 and above	105 (24.9)	56 (31.8)	49 (20.0)	
Refused to answer	69 (16.4)	31 (17.6)	38 (15.5)	
**Household dynamics**				
Dual-parent	383 (89.1)	164 (90.6)	219 (87.9)	0.384
Single-parent	47 (10.9)	17 (9.4)	30 (12.1)	
**Diabetes duration**				
≤ 4 years	203 (49.4)	82 (46.3)	121 (51.7)	0.280
>4 years	208 (50.6)	95 (53.7)	113 (48.3)	
*Median (IQR)*	4 (2–6)	4.21 (2.6–6.8)	3.8 (2.1–5.9)	0.106
**HbA1c**				
≤ 7.0%	12 (5.0)	2 (1.9)	10 (7.3)	0.018^*^
7.0% ≤ 9.0%	65 (27.1)	22 (21.2)	43 (31.6)	
>9.0%	163 (67.9)	80 (76.9)	83 (61.0)	
*Median (IQR)*	9.7 (8.4–11.3)	10.1 (9.1–11.6)	9.4 (8.2–10.8)	< 0.01^*^
**Insulin regimen**				
MDI	380 (85.6)	162 (85.7)	218 (85.5)	0.947
CSII	64 (14.4)	27 (14.3)	37 (14.5)	
**T1D family history** ^**^				
Yes	108 (24.8)	45 (24.6)	63 (24.9)	0.941
No	328 (75.2)	138 (75.4)	190 (75.1)	

T1D, type 1 diabetes; IQR, interquartile range; KD, Kuwaiti Dinar; HbA1c, Hemoglobin A1c; MDI, multiple daily injections; CSII, continuous subcutaneous insulin infusion; HRQoL, Health-Related Quality of Life; COVID-19, coronavirus disease of 2019.

* p < 0.05.

** Defined as a participant having a first-degree relative diagnosed with Type 1 diabetes.

Table 2 describes the self-reports and parent-proxy reports of total and domain-specific HRQoL scores of the whole study population. The mean ± SD total HRQoL score of the self-report was significantly higher compared with those of the parent-proxy reports (79.06 ± 15.19 vs. 73.79 ± 15.17, *p* < 0.01). Children reported significantly higher scores than their parents in the “treatment barriers I” and “worry” domains (*p* < 0.01). However, children reported a significantly lower score (62.59 ± 18.04) in the “diabetes” symptoms domain than their parent-proxy reports (70.06 ± 19.66, *p* < 0.01).

**Table 2:** HRQoL of self-reports and parent-proxy reports during the COVID-19 pandemic.

	**Total**			**Affected by COVID-19**			**Unaffected by COVID-19**		
	**Self-report**	**Parent-proxy**	***p***	**Self-report**	**Parent-proxy**	***p***	**Self-report**	**Parent-proxy**	***p***
	**(*****n*** = **165)**	**(*****n*** = **455)**		**(*****n*** = **69)**	**(*****n*** = **191)**		**(*****n*** = **96)**	**(*****n*** = **264)**	
**Peds QL**^TM^ **Diabetes Module domains**									
	**M** **±SD**	**M** **±SD**		**M** **±SD**	**M** **±SD**		**M** **±SD**	**M** **±SD**	
Total	79.06 ± 15.19	73.79 ± 15.17	< 0.01^*^	77.04 ± 15.81	72.80 ± 14.90	0.047^*^	80.51 ± 14.65	74.50 ± 15.36	< 0.01^*^
Diabetes	62.59 ± 18.04	70.06 ± 19.66	< 0.01^*^	59.50 ± 17.69	68.30 ± 20.16	< 0.01^*^	64.93 ± 18.05	71.34 ± 19.22	< 0.01^*^
Treatment I	82.28 ± 20.81	75.09 ± 24.84	< 0.01^*^	81.43 ± 24.28	72.05 ± 26.12	< 0.01^*^	82.92 ± 17.86	77.30 ± 23.67	0.039^*^
Treatment II	83.23 ± 19.66	83.99 ± 18.67	0.660	82.58 ± 16.94	82.08 ± 20.08	0.856	83.70 ± 21.49	85.38 ± 17.48	0.454
Worry	73.48 ± 33.28	61.98 ± 41.88	< 0.01^*^	69.71 ± 36.63	65.20 ± 40.04	0.418	76.39 ± 30.35	59.65 ± 43.10	< 0.01^*^
Communication	82.15 ± 26.43	78.64 ± 29.82	0.187	81.40 ± 26.74	76.63 ± 30.29	0.251	82.71 ± 26.33	80.06 ± 29.46	0.446

Peds QL^TM^, The Pediatric Quality of Life Inventory (Peds QLTM) Diabetes Module 3.0.

* p < 0.05.

Higher scores indicate better HRQoL.

M, mean; SD, standard deviation; HRQoL, Health-Related Quality of Life; COVID-19, Coronavirus disease of 2019.

In the COVID-19-affected group, there was a significant difference between the total self-reported HRQoL (77.04) and the parent-proxy HRQoL score (72.80) (*p* = 0.047). The COVID-19-affected children reported a significantly lower HRQoL score in the “diabetes” symptoms domain in comparison to parent-proxy reports (59.50 vs. 68.30, *p* < 0.01). However, they reported a significantly higher score in the “treatment barriers I” domain (81.43) than their parent-proxy reports (72.05) (*p* < 0.01).

Similar to the COVID-19-affected group, children unaffected by COVID-19 reported higher total HRQoL scores (80.51) compared with their parents (74.50) (*p* < 0.01). Furthermore, parent-proxies in the unaffected COVID-19 group consistently reported significantly lower scores compared with self-reports in the “treatment barriers I” domain (*p* = 0.039). The children unaffected by COVID-19 also reported a significantly lower score in the “diabetes” symptoms domain than their parent-proxy reports, similar to the COVID-19 affected group (64.93 ± 18.05 vs. 71.34 ± 19.22, *p* < 0.01).

Table 3 compares the self-report and parent-proxy reports of the affected and unaffected groups (by COVID-19). There was no significant difference between the two groups with regard to self-reports in total or domain-specific scores. However, the parents of COVID-19-affected children reported a significantly lower score in the “treatment barriers I” domain than the unaffected group (72.05 ± 26.12 vs. 77.30 ± 23.67, *p* = 0.026). Although marginally significant (*p* = 0.068), parents in the COVID-19-affected group reported lower scores in the “treatment adherence II” domain compared with parents unaffected by COVID-19 (82.08 ± 20.08 vs. 85.38 ± 17.48, *p* = 0.068).

**Table 3:** Comparison of self-reports and parent-proxy reports for those affected vs. unaffected by COVID-19.

	**Self-report**			**Parent-proxy report**		
	**Affected by COVID-19**	**Unaffected by COVID-19**	***p-value***	**Affected by COVID-19**	**Unaffected by COVID-19**	***p-value***
**Peds QL**^TM^ **Diabetes Module domains**						
	**M** **±SD**	**M** **±SD**		**M** **±SD**	**M** **±SD**	
Total	77.04 ± 15.81	80.51 ± 14.65	0.149	72.80 ± 14.90	74.50 ± 15.36	0.238
Diabetes	59.50 ± 17.69	64.93 ± 18.05	0.059	68.30 ± 20.16	71.34 ± 19.22	0.104
Treatment I	81.43 ± 24.28	82.92 ± 17.86	0.655	72.05 ± 26.12	77.30 ± 23.67	0.026^*^
Treatment II	82.58 ± 16.94	83.70 ± 21.49	0.720	82.08 ± 20.08	85.38 ± 17.48	0.068
Worry	69.71 ± 36.63	76.39 ± 30.35	0.218	65.20 ± 40.04	59.65 ± 43.10	0.164
Communication	81.40 ± 26.74	82.71 ± 26.33	0.757	76.63 ± 30.29	80.06 ± 29.46	0.232

Peds QL^TM^, The Pediatric Quality of Life Inventory (Peds QLTM) Diabetes Module 3.0.

* p < 0.05.

Higher scores indicate better HRQoL.

M, mean; SD, standard deviation; HRQoL, Health-Related Quality of Life; COVID-19, coronavirus disease of 2019.

## 4. Discussion

This study aimed to evaluate the HRQoL of children and adolescents with T1D based on parent-proxy reports using the PedsQL™ 3.0 Diabetes module during the COVID-19 pandemic. This information may provide insights into how experiences of disease management during the COVID-19 pandemic may have been perceived differently by children and their parents. We further compared HRQoL reports from children and parent-proxies who had been affected with COVID-19 infection (personal or familial) with those unaffected.

We found that, regardless of COVID-19 infection status, children and adolescents with T1D reported higher scores (indicating better HRQoL) in the total HRQoL, “treatment barriers I,” and “worry” domains, compared with their parents. Furthermore, children and adolescents in both groups reported lower scores in the “diabetes symptoms” domain, indicating poorer HRQoL. In those affected by COVID-19, parents reported significantly worse HRQoL in “treatment barriers I” and marginally worse HRQoL in the “treatment adherence II” domain, compared with parents whose children were unaffected by COVID-19 infection.

Regardless of being affected or unaffected by COVID-19, parents of children with T1D during the pandemic reported worse HRQoL than what children reported. This finding is consistent with other studies published before the pandemic on children who suffer from T1D and obesity in Kuwait, and children with diabetes in Saudi Arabia (16, 22, 23). In Kuwait, Abdul-Rasoul et al. found that parents of children with T1D reported worse HRQoL than their children (16). Studies in Italy and Norway also showed that parents of children with T1D perceived a lower total quality of life compared with their children (20, 24). This might be attributed to the burden of the disease, the complexity of management, and concern for their child's lifestyle and future (16, 23, 25). Our study demonstrated that such discrepancy between the parents' reports and their children's reports on HRQoL continues to exist during the pandemic. It is therefore warranted to suggest incorporating an assessment of HRQoL during a pandemic to provide support, if needed, by facilitating open communication between children and their families, identifying points of concern from both sides, and planning for joint intervention.

In both groups, parent and child perception of diabetes symptoms, treatment barriers and adherence, worry about sleep quality, anger management, and hypoglycemic episodes were significantly different, consistent with the findings from Kuwait, Saudi Arabia, and Italy (16, 23, 24). The discrepancy in several of the domains indicates the importance of collecting information from the viewpoint of both child and parent, to better understand the impact of disease on HRQoL. The reduced quality of life in the “diabetes symptoms” domain in the children's report may be attributed to more personal effects of diabetes on the child's experiences with the disease.

This study also found that COVID-19-affected parents experienced worse HRQoL in the “treatment barrier” domain, compared with their unaffected counterparts. Treatment barriers include, for example, feeling pain when getting a finger prick or taking an insulin shot, feeling embarrassed about treatment, arguing with parents, or difficulty in caring for their diabetes. To this end, this is the first report that evaluates HRQoL between COVID-19-affected and unaffected children with T1D. Parents' added fear and worry regarding COVID-19 infection in the child or a family member adds to the challenges of managing diabetes and compliance with therapy during such stressful periods (26). The parental burden of management of a chronic condition such as T1D could also be amplified due to the lack of knowledge in relation to dealing with T1D during the pandemic, especially when infected with the infection itself (24–26). Infection control measures implemented by health authorities has led to limited interactions between children with T1D and their healthcare providers, which might indeed contribute to the lack of guidance during the pandemic period (27).

Our study is one of the first reports on HRQoL in children with T1D during the COVID-19 pandemic, regionally or internationally. To conduct the study, we used a validated translated questionnaire, with a disease-specific module, to assess the HRQoL of children and adolescents. There are a number of limitations in the current study. We can only generalize to Arabic speakers in Kuwait, as the data collection instruments were available in Arabic for the current study. The study was cross-sectional; therefore, it cannot provide information on changes in HRQoL over time, or before or after being affected by COVID-19.

## 5. Conclusion

This is the first report on the health-related quality of life of children with T1D in Kuwait during the COVID-19 era. Parents' or caregivers' experience of caring for their children was negative, as they worried, and reported poorer HRQoL compared with their children's perceptions. There is a need to empower healthcare professionals to support parents and caregivers of children with chronic diseases such as T1D in promoting self-management, enhancing physical and psychological wellbeing, treatment adherence, and continuous health education during pandemics of any kind. Longitudinal studies on the effect of the COVID-19 pandemic on children and youth with T1D are necessary to enhance our understanding of the effects of the pandemic on disease management, glycemic control, day-to-day activities, and patients' wellbeing. Future studies could also add new insights by comparing the experiences of children and adolescents with and without diabetes.

## Data availability statement

The original contributions presented in the study are included in the article/supplementary material, further inquiries can be directed to the corresponding author.

## Ethics statement

The studies involving human participants were reviewed and approved by Ethical Committee at the Ministry of Health of Kuwait. Written/verbal consents were provided by legal guardians or next of kin.

## Author contributions

DA-A was responsible for planning, designing, data managing, conducting the study, and writing the manuscript. TA, FO, and FA-J collected data and recruited patients. DK participated in directing the reported work, data management, critically reviewed the manuscript, and participated in the discussions. TA, DA-A, and DK carried out data analysis and interpretation of the results. TA, FO, SQ, and FA-J contributed to the writing of the manuscript. HA-K participated in the planning, data management, and conducting of the study. All authors contributed to the article and approved the submitted version.
